# Development and validation of a novel bioassay to determine glucocorticoid sensitivity

**DOI:** 10.1186/s40364-016-0079-y

**Published:** 2016-12-15

**Authors:** Emily L. Williams, Madeleine L. Stimpson, Peter L. Collins, Doyo G. Enki, Ashish Sinha, Richard W. Lee, Ashwin D. Dhanda

**Affiliations:** 1 School of Clinical Sciences, Medical Sciences Building, University of Bristol, Bristol, BS9 1TD UK; 2 National Institute for Health Research Biomedical Research Centre at Moorfields Eye Hospital NHS Foundation Trust and UCL Institute of Ophthalmology, University Hospitals Bristol NHS Foundation Trust and University of Bristol, Bristol, UK; 3 Department of Liver Medicine, University Hospitals Bristol NHS Foundation Trust, Bristol, BS2 8HW UK; 4 Biostatistics, Bioinformatics and Biomarkers research group, Plymouth University, N15 Plymouth Science Park, Plymouth, PL6 8BX UK; 5 Institute of Translational and Stratified Medicine, Plymouth University Peninsula Schools of Medicine and Dentistry, John Bull Building, Plymouth, PL6 8BU UK; 6 South West Liver Unit, Plymouth Hospitals NHS Trust, Plymouth, UK

**Keywords:** Glucocorticoid sensitivity, Biomarker, Proliferation, Bromodeoxyuridine

## Abstract

**Background:**

Glucocorticoids (GCs) remain the first line treatment for almost all non-infectious inflammatory diseases, ranging from acute asthma to rheumatoid arthritis. However, across all conditions, patients have a variable response to GCs with approximately 30% being non-responders. This group of GC resistant patients is typically exposed to high-dose GCs and their side-effects before more appropriate immunotherapy is instituted. Hence, there is a pressing clinical need for a predictive biomarker of GC responsiveness. The availability of such a tool would also enable patient stratification for the conduct of smart clinical trials in GC resistance. Lymphocyte GC sensitivity has been shown to be closely associated with clinical GC sensitivity in a number of inflammatory diseases. However, the method for determining in vitro GC response is not standardized and requires the use of specialist equipment, including a radioisotope to quantify cellular proliferation, making it challenging to translate into clinical practice.

**Results:**

Here we describe the optimization and validation of a novel non-radioactive in vitro bioassay based on measuring cellular proliferation by incorporation of bromodeoxyuridine (BrdU), termed the BrdU incorporation in lymphocyte steroid sensitivity assay (BLISS). In comparison to the current gold standard lymphocyte GC sensitivity assay in 101 healthy control samples, BLISS has an area under receiver operating characteristic of 0.82 and a sensitivity of 83% for correctly identifying GC resistant subjects.

**Conclusions:**

The performance of the novel BLISS bioassay makes it a strong candidate biomarker for clinical application. It now requires validation in a prospective patient cohort.

**Electronic supplementary material:**

The online version of this article (doi:10.1186/s40364-016-0079-y) contains supplementary material, which is available to authorized users.

## Background

For more than half a century glucocorticoids (GCs) have formed the mainstay of the initial treatment of almost all non-infectious inflammatory diseases including asthma, inflammatory bowel disease, rheumatoid arthritis, non-infectious uveitis and acute severe alcoholic hepatitis. However, response to GCs in patient populations is variable and in each disease group a cohort of GC non-responders (approximately 30% of all patients) has been clearly described [1–4]. GC resistant patients are both at risk of the consequences of their uncontrolled disease and, because they require higher and longer courses of therapy, they also incur a greater burden of treatment side-effects. This could be avoided if alternative immunotherapeutics were to be instituted early in their disease course, but at present there is no way of identifying GC resistant individuals prior to GC treatment. Hence, there is an overt clinical need to develop a biomarker of GC resistance prior to induction of therapy as a tool for personalized healthcare.

GCs have wide ranging effects on many cells and tissues. They bind to cytoplasmic GC receptors, which translocate to the nucleus and bind to DNA at GC responsive elements resulting in transcriptional regulation by either transactivation or transrepression of target genes, leading to reduction in inflammation. The main mechanism of GC anti-inflammatory action is through interference with pro-inflammatory transcription factors such as nuclear factor kappa B and activator protein 1 [5]. Although efficacious in many diseases, prolonged exposure to GCs results in numerous undesirable side-effects including osteoporosis, diabetes, infection and increased cardiovascular risk [6, 7]. Therefore GCs should be used selectively in responsive patients where a therapeutic benefit can be achieved. Measurement of individual GC sensitivity could therefore be crucial to permit appropriate stratified treatment.

In patients with bronchial asthma it was demonstrated that GC sensitivity correlated with in vitro lymphocyte GC sensitivity rather than with a measure of disease severity, decline in lung function [8, 9]. In these studies, GC sensitivity was quantified in vitro by measuring the inhibitory effect of dexamethasone (a synthetic GC) on phytohaemagluttinin (PHA)-induced lymphocyte proliferation in peripheral blood mononuclear cells (PBMCs). This assay, known as the Dexamethasone Inhibition of Lymphocyte Proliferation Assay (DILPA), demonstrates a wide range in GC sensitivity in healthy controls, which is stable over time and not related to intrinsic cortisol production [10]. Approximately 30% of the reported population, and also in other healthy control populations, are classified as GC resistant by DILPA [10, 11], which corresponds to the overall proportion of GC resistant patients. This suggests that the individual’s GC sensitivity is inherent in the presence or absence of disease. When applied to inflammatory diseases DILPA predicts clinical GC response better than traditional measures of disease severity in patients with ulcerative colitis [12, 13], alcoholic hepatitis [3, 14] and rheumatoid arthritis [15].

Traditionally, in order to quantify lymphocyte GC sensitivity, cell proliferation is determined by labelling dividing cells with tritiated thymidine (^3^H, a radioisotope of hydrogen) and measuring the beta radiation emission using a beta counter. This technique necessitates expensive equipment that is not widely available in many healthcare or commercial laboratories. Alongside this, there are stringent health and safety standards that can impede the use of radiation for many organizations. The clinical translation of this assay has therefore been limited and currently only a few institutions are performing the DILPA in a research setting.

There is consequently a requirement for an alternative GC sensitivity assay which can be easily performed in any laboratory setting. Therefore, the aim of this study was to develop and validate a novel bioassay to measure in vitro GC sensitivity. Bromodeoxyuridine (BrdU) is a synthetic pyrimidine analogue of thymidine, which is incorporated into the DNA of cells undergoing division in the S phase of the cell cycle and can be detected using labelled antibodies to BrdU by chemiluminescence, flow cytometry or colorimetric assays. Therefore, detection of newly synthesized DNA using BrdU can be used as an indirect measure of cell proliferation [16–18]. Using a commercially available colorimetric assay, which does not require bespoke equipment or expertise, we have developed a novel measure of GC sensitivity termed the BrdU Lymphocyte Incorporation Steroid Sensitivity (BLISS) assay. In this report we describe the full assay protocol and validation of the BLISS assay to measure clinical GC sensitivity in comparison to the standard DILPA.

## Methods

This study was conducted under ethical approval from the NHS Health Research Authority (reference: 04/Q2002/84). After obtaining written informed consent 20 mL peripheral blood was taken from healthy subjects by standard venepuncture into EDTA collection tubes. PBMCs were isolated by density gradient centrifugation over Ficoll-Paque Plus (VWR International, Lutterworth, Leicestershire, UK) using Leucosep tubes (Greiner Bio-One International, Stonehouse, Gloucestershire, UK) and cell viability assessed by Trypan blue exclusion. Cells were counted manually by light microscopy using a hemocytometer.

### DILPA

The DILPA was performed as previously described [3, 12]. In brief, 2 × 10^6^ PBMCs / ml were suspended in RPMI media (Thermo Fisher Scientific, Loughborough, Leicestershire, UK) supplemented with 10% heat inactivated fetal calf serum (Thermo Fisher Scientific), 2 mM L-glutamine (Thermo Fisher Scientific) and 100 U/mL penicillin and streptomycin (Thermo Fisher Scientific). Cells were cultured in a 96-well plate in triplicate with 20 μg/mL PHA (Sigma-Aldrich, Poole, Dorset, UK) in the presence or absence of 1 × 10^−6^ M Dexamethasone (Sigma-Aldrich, Poole, Dorset) for 42 h at 37^°^C with 5% CO_2._ A saturating dose of 185 kBq/ml ^3^H was then added and cultured for a further 6 h. Control wells with unstimulated cells were also included. The plate was harvested onto glass fibre filter paper (Wallac Oy, Turku, Finland) using a cell harvester (Skatron, Cox Scientific, Rothwell, Northamptonshire, UK) and beta emission measured using a Micro β emission scintillation counter (Wallac) in counts per minute (cpm). Samples with cpm < 10,000 were considered a technical failure and excluded from analysis (*n* = 6 in the optimization phase of experiments).

### BLISS assay

PBMCs were isolated and cultured for 42 h in triplicate as described for the DILPA. Control wells of media alone, BrdU reagent alone and anti-BrdU-peroxidase antibody alone were also included in triplicate. BrdU reagent was added for the final 6 h of culture at 10 μM. BrdU was then detected using a commercial kit according to the manufacturer’s instructions (Roche Diagnostics, Burgess Hill, UK). The protocol was optimized to determine the most appropriate cell density, processing time and time of exposure to the developing substrate. The final standardized protocol is described fully in the results section.

### Statistical analysis

For the standard DILPA and BLISS assay the maximum percentage inhibition of proliferation (Imax) was calculated:

$$ \mathrm{Imax} = \left(1\ \hbox{--}\ \left[\mathrm{proliferation}\ \mathrm{in}\ \mathrm{presence}\ \mathrm{of}\ \mathrm{G}\mathrm{C}\right]\ /\ \left[\mathrm{proliferation}\ \mathrm{in}\ \mathrm{absence}\ \mathrm{of}\ \mathrm{G}\mathrm{C}\right]\right) \times 100 $$

Although Imax values for both DILPA and BLISS give a percentage suppression of proliferation by GCs, the underlying assays use different methods with different units and scales. Therefore, in order to place both assays on a similar scale to compare the results more meaningfully, the BLISS dataset was first calibrated using an inverse regression model.

A Bland and Altman test of agreement was then performed between DILPA and the adjusted BLISS and 95% limits of agreement were determined [19].

To assess the accuracy of the adjusted BLISS Imax in predicting DILPA Imax an area under receiver operating characteristic (AUROC) was calculated and compared to the diagonal reference line.

The previously determined threshold to define GC sensitivity of Imax = 60% [10] was applied to the DILPA dataset. The 60% threshold was adjusted according to the inverse regression model and applied to the BLISS dataset. Accuracy of the BLISS assay was compared to the DILPA applying the adjusted threshold to produce categorical data to give a contingency table.

## Results

### Subjects

Following optimization, 101 assays were performed on samples from unique healthy individuals using the standardized BLISS assay technique as described below.

There were no DILPA values excluded from the final analysis due to technical failure with low proliferation in PHA stimulated wells < 10,000 cpm. For the BLISS assay there was a single technical failure for sample with low proliferation in the PHA stimulated wells which was not included in the final analysis.

### Standardized BLISS assay protocol

PBMCs were cultured at 2 × 10^6^ / ml in triplicate in a 96-well plate exactly as described for the standard DILPA with 20 μg/ml PHA in the presence or absence of 1 × 10^−6^ M dexamethasone at 37^°^C with 5% CO_2_ for 42 h in a total volume of 200 μL. Twenty microlitre per well BrdU labelling solution diluted 1:100 in RPMI supplemented with 10% FCS, L-glutamine and antibiotics (complete media) were added to all experimental wells and to media alone for a negative control and cultured for a further 6 h. Cells were resuspended and the entire well volume transferred directly to a 96-well flat bottomed plate and centrifuged for 10 min at 300 g. Supernatants were aspirated and discarded. The plate was dried for 1 h in a 60^°^C oven or for 15 min using a standard hair drier. The dry plate was covered and left overnight at 4^°^C. For more rapid results this step can be omitted by proceeding directly to fixing the cells and denaturing the DNA. Results are similar if processed directly or after keeping at 4^°^C overnight (Pearson’s r = 0.82 for 29 subjects). 200 μL / well of FixDenat solution was added to fix and denature the DNA and incubated for 30 min at room temperature. FixDenat solution was removed by flicking off and tapping plate. 100 μL / well anti-BrdU-peroxidase conjugate (pre-diluted at 1:100 with antibody diluent solution) was added to all experimental wells and to negative control wells. The plate was incubated for 90 min at room temperature. Antibody solution was removed by flicking off and tapping plate. Plates were washed three times with 200 μL / well of washing solution diluted 1:10 with distilled H_2_O. A final wash was performed with 200 μL / well Phosphate Buffered Saline (PBS). Wash solution was removed by flicking off and tapping plate. 100 μL / well of substrate was added and incubated for 5 min at room temperature. Absorbance of the samples was measured in an ELISA plate reader at 370 nm (reference wavelength 492 nm). Absorbance (A_370nm_–A_492nm_) was calculated for each well. The value for Imax was then also calculated as shown above.

### BLISS and DILPA data have different distributions

**Fig. 1:**
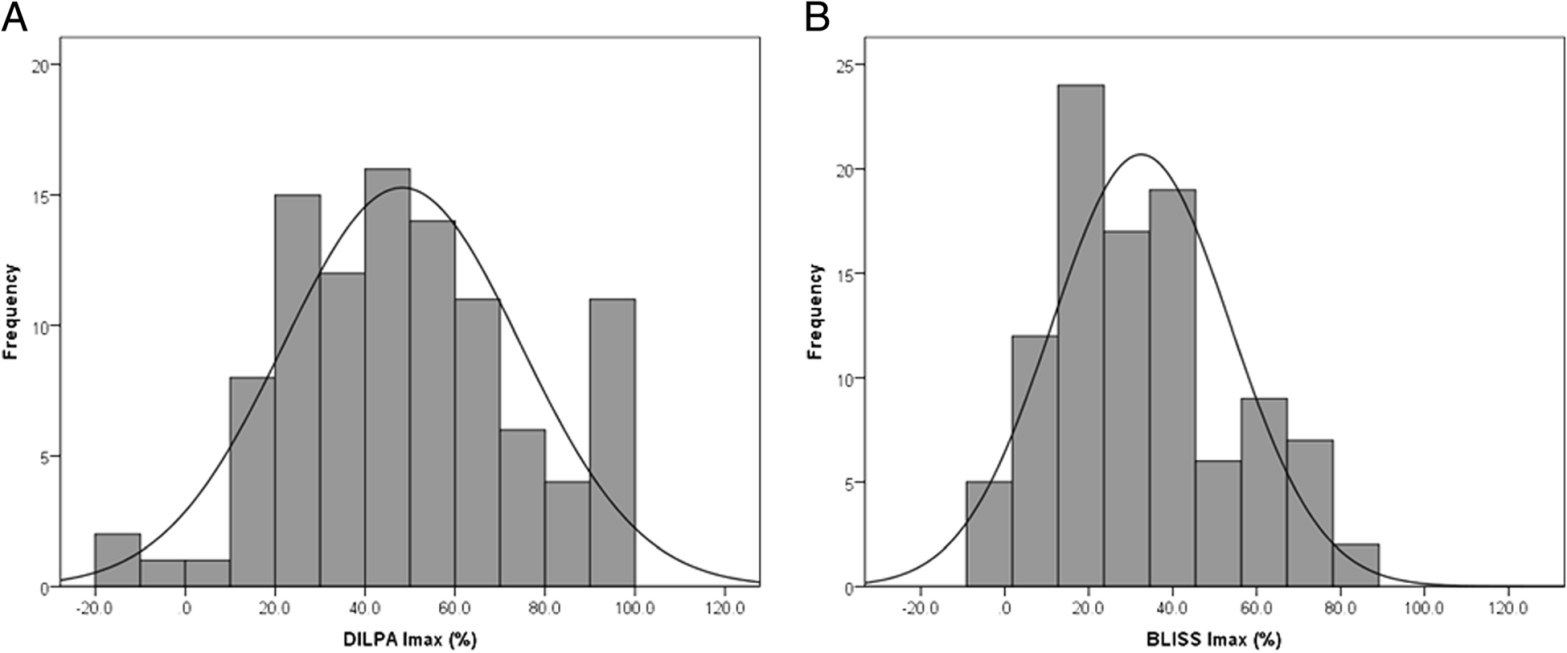
Distribution of Imax for DILPA **a** and BLISS assay **b**. Frequencies have been binned in 10% groups in 101 healthy control samples and the normal distribution curve overlaid. The mean and standard deviation for DILPA is 48% and 26 and for BLISS 32% and 21, respectively. Their distributions are significantly different (unpaired *t*-test *p* < 0.001)

The Imax values for both assays are approximately normally distributed, confirming that there is a range of GC sensitivity seen in the healthy population (Fig. 1). However, the mean and standard deviations (sd) for each test are significantly different: mean DILPA is 48.2% (sd 26.3%) and mean BLISS is 32.5% (sd 21.2%) (*p* < 0.001; unpaired *t* test).

### BLISS assay accurately predicts GC resistance

Inverse regression provides the following model:

$$ \mathrm{BLISS}\ \mathrm{Imax} = 6.8 + \left(0.53 \times \mathrm{DILPA}\ \mathrm{Imax}\right) $$

**Fig. 2:**
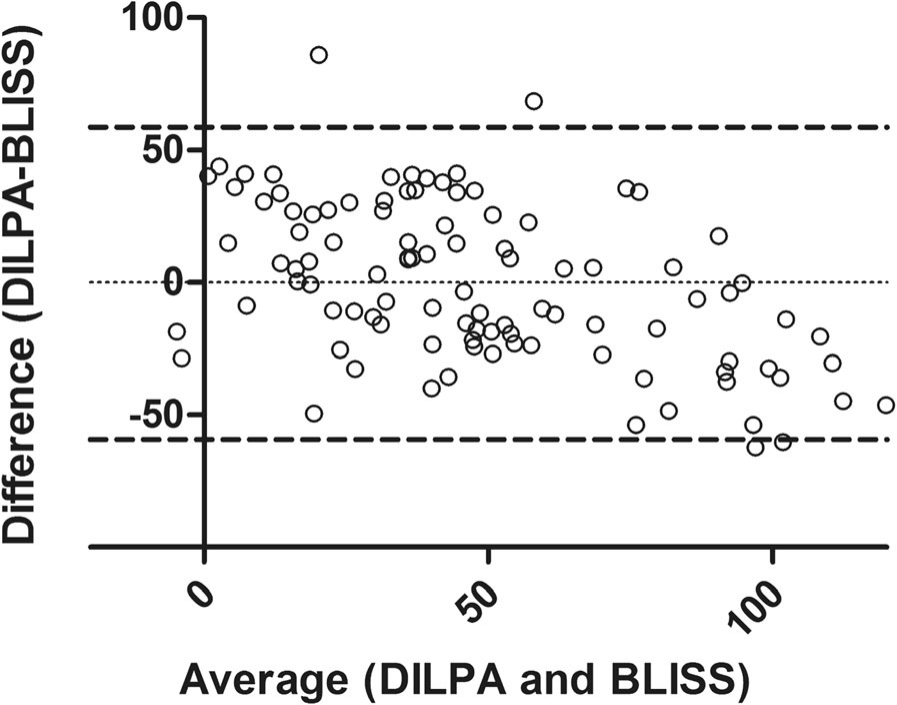
Bland and Altman test of agreement for DILPA and BLISS. Bland and Altman plot of average versus difference for DILPA and BLISS (adjusted by the inverse regression model of 6.8 + [5.3 × DILPA]). Bias (mean difference between DILPA and BLISS) is −0.2% with 95% limits of agreement from −59.2 to 58.7% (*dashed lines*)

The BLISS dataset was then adjusted by using the equation (BLISS Imax–6.8) / 0.53 and a Bland and Altman test of agreement was performed (Fig. 2). The bias was found to be −0.2% with 95% limits of agreement from −59.2 to 58.7%.

**Fig. 3:**
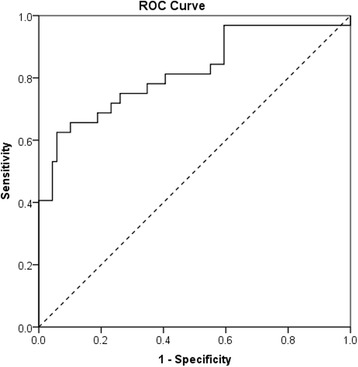
BLISS has a high accuracy in predicting DILPA. Receiver operating characteristic for BLISS (adjusted by inverse regression model) in predicting DILPA with diagonal reference line (*dashed*). AUROC = 0.82 (95% CI 0.73–0.92; *p* < 0.001 compared to reference line)

Using the adjusted BLISS dataset to predict DILPA, AUROC was 0.82 (95% confidence interval [CI]: 0.73–0.92; *p* < 0.001; Fig. 3).

Applying the inverse regression model to the predefined threshold for GC sensitivity of 60% for DILPA [12] gives a threshold of 38.7% for BLISS. Classifying subjects as GC sensitive or GC resistant using the predefined threshold for the DILPA and the adjusted threshold for BLISS demonstrates that the BLISS assay has 83% sensitivity (95% CI: 71–90%) and 69% specificity (95% CI: 50–83%) compared to the DILPA (Table 1).

**Table 1:** Contingency table comparing classification of GC sensitive (GC-S) and resistant (GC-R) subjects by DILPA and BLISS assays

		DILPA	
		GC-R (Imax < 60%)	GC-S Imax > 60%)
BLISS	GC-R (Imax < 39%)	57	10
	GC-S (Imax > 39%)	12	22

Sensitivity: 83% (95% confidence interval 71–90%)

Specificity: 69% (50–83%)

Positive Predictive Value: 85% (74–92%)

Negative Predictive Value: 65% (46–80%)

## Discussion

Here we report the use of the novel BLISS assay to determine GC sensitivity. It has a suitably high AUROC (0.82) and sensitivity in correctly identifying GC non-responders (83%) in reference to the existing gold standard lymphocyte GC sensitivity assessment method (DILPA). The sensitivity of BLISS in identifying GC non-responders is the most important measure of diagnostic accuracy; it must correctly identify those patients who do not respond to GCs so as to reliably reduce their unnecessary exposure to GCs and guide alternative management. Secondly, it is important to identify those who can benefit from GCs so as not to inappropriately withhold GC treatment. Therefore specificity of BLISS is of secondary importance (69% in comparison to DILPA).

The simplicity of the assay, which uses a commercially available kit and does not require complex equipment or processing techniques, means it can be performed in any laboratory that has cell culture facilities. Unlike the DILPA, the BLISS assay does not have any radiation safety concerns and does not require the purchase and maintenance of expensive laboratory equipment. The BLISS assay is also more reliable than the DILPA. In 10 subjects who had DILPA and BLISS repeated on a second occasion several weeks later, there was poor correlation with their previous DILPA Imax value (r = 0.45). The BLISS assay demonstrated closer correlation (r = 0.59). In addition, there was also <1% technical failure rate. These attributes make this an appealing assay to apply to patients with a range of inflammatory and autoimmune diseases with potential to allow clinicians to stratify patient risk and develop more individualized treatment programs.

We note that the Bland and Altman test of agreement demonstrated wide 95% limits of agreement between the DILPA and BLISS assays. This is likely to reflect the variability of functional assays and particularly the measurement of proliferation by ^3^H incorporation and beta emission detection. However, the Bland and Altman test of agreement is not an appropriate statistical measure for comparison between the DILPA and BLISS since, although readings are converted to the same units (Imax), the underlying quantities differ (cpm and absorbance) [20]. Therefore, regression together with AUROC analysis and a categorical comparison are more appropriate to determine whether the BLISS assay is similar to the DILPA in identifying GC resistant patients. Additionally, in a cohort of healthy controls in the absence of disease it is impossible to correlate either assay with clinical phenotype and steroid responsiveness. Therefore the BLISS assay now requires validation in a cohort of patients with inflammatory diseases. Such validation is currently underway in a cohort of patients with acute severe alcoholic hepatitis, an inflammatory disease of the liver in which GCs are the only treatment known to improve survival. Preliminary data from 10 patients demonstrate that the BLISS assay significantly correlates with the current gold standard measure of GC responsiveness known as the Lille score with a correlation coefficient of −0.74 (*p* = 0.02; Additional file 1: Table S1) [21]. The Lille score is calculated from clinical and biochemical parameters after 7 days of GC treatment with higher Lille scores associated with greater GC resistance (hence the negative correlation coefficient). This suggests that the BLISS assay has clinical translatability which will be addressed after the adequately powered study in 200 patients has been completed.

In this normal volunteer cohort we applied an adjusted GC sensitivity threshold to the BLISS assay based on the inverse regression model. However, this may not be appropriate because there is a different distribution of BLISS assay Imax with a lower mean and standard deviation than the DILPA. Accurate determination of the best threshold to define GC sensitivity is best achieved in a new cohort of patients with clear clinical definitions of GC sensitivity and resistance, which are independent of the assay.

In current clinical practice, GC responsiveness is determined by changes in clinical symptoms or disease activity scores specific to the condition, which can take several days to respond. For example, in patients with acute severe alcoholic hepatitis GC response is determined at day 7 of treatment according to the Lille score [21]. Although the BLISS assay represents a significant improvement in the speed of determining clinical GC responsiveness, results still take a little over 48 h to obtain. This may be relevant in certain conditions in which urgent GC treatment cannot be delayed (e.g. acute severe ulcerative colitis or acute life-threatening asthma). However, harm to the patient from a short period of GC treatment is unlikely and the BLISS assay will still have clinical utility to guide subsequent patient management such as escalating therapy in GC resistant patients.

Alternative methods have been reported to measure cell proliferation by BrdU incorporation including chemiluminescence and flow cytometry [17, 22]. The chemiluminescence method is similar to the colorimetric assay but uses a different substrate with 2 components and requires a luminometer with photomultiplier to detect light emission. Our proposed protocol uses a premixed substrate and ELISA plate reader to measure wavelength absorbance, making it marginally easier to apply. Flow cytometry methods for BrdU measurement allow quantification at a single cell level. However, flow cytometry requires the appropriate expertise and equipment and may struggle with high throughput, limiting its generalizability and translation into clinical laboratories. The colorimetric assay we describe here is therefore the most streamlined and easily accessible technique to measure cell proliferation.

## Conclusions

In summary, we have reported the validation of a simple novel bioassay with a standardized laboratory protocol which accurately measures GC sensitivity. This has broad translational implications and could be applied to many inflammatory diseases to guide clinical management of individual patients, ensuring that GC responsive patients are correctly treated with GCs while reducing unnecessary exposure to GCs and accelerating the appropriate escalation of treatment of GC resistant patients. Further validation in a clinical context is ongoing.

## Additional file

Additional file 1: BLISS correlates with clinical GC sensitivity in patients with acute severe alcoholic hepatitis. Preliminary data in 10 patients with acute severe alcoholic hepatitis demonstrates significant correlation with the Lille score (based on clinical and biochemical parameters after 7 days of GC treatment). Lille scores range from 0 to 1 with higher Lille scores associated with greater clinical GC resistance. Correlation coefficient = −0.74; *p* = 0.02 (DOCX 14 kb)

## Abbreviations

AUROC: Area under receiver operating characteristic
BLISS: BrdU in lymphocyte incorporation steroid sensitivity assay
BrdU: Bromodeoxyuridine
DILPA: Dexamethasone inhibition of lymphocyte proliferation assay
GC: Glucocorticoid
Imax: Maximum inhibition of proliferation with dexamethasone
PBMC: Peripheral blood mononuclear cell
PHA: Phytohaemagluttinin
