# Supplementary material for: Development and validation of a novel bioassay to determine glucocorticoid sensitivity
Source: Biomark Res. 2016 Dec 15;4:26. doi: 10.1186/s40364-016-0079-y (PMC5157083; doi:10.1186/s40364-016-0079-y)
Supplement: Additional file 1: — BLISS correlates with clinical GC sensitivity in patients with acute severe alcoholic hepatitis. Preliminary data in 10 patients with acute severe alcoholic hepatitis demonstrates significant correlation with the Lille score (based on clinical and biochemical parameters after 7 days of GC treatment). Lille scores range from 0 to 1 with higher Lille scores associated with greater clinical GC resistance. Correlation coefficient = −0.74; p = 0.02 (DOCX 14 kb) [file 40364_2016_79_MOESM1_ESM.docx]

**Supplementary Table**

| **Patient ID** | **Lille score** | **BLISS Imax (%)** |
| --- | --- | --- |
| 001 | 0.08 | 16 |
| 002 | 0.09 | 3 |
| 003 | 0.03 | -4 |
| 004 | 0.09 | 26 |
| 005 | 0.23 | -31 |
| 006 | 0.02 | 15 |
| 007 | 0.29 | 29 |
| 008 | 0.75 | -61 |
| 009 | 0.08 | 37 |
| 010 | 0.05 | 9 |

BLISS correlates with clinical GC sensitivity in patients with acute severe alcoholic hepatitis. Preliminary data in 10 patients with acute severe alcoholic hepatitis demonstrates significant correlation with the Lille score (based on clinical and biochemical parameters after 7 days of GC treatment). Lille scores range from 0 to 1 with higher Lille scores associated with greater clinical GC resistance. Correlation coefficient = -0.74; p=0.02.
